# Ernest Hart, D.C.L., M.R.C.S.: An Appreciation

**Published:** 1898-01-15

**Authors:** 


### Ernest Hart, D.C.L., M.R.C.S.: An Appreciation.

(By an Old Friend and Colleague.

It is, I fear, true that the men born to do the work of the world are almost necessarily unpopular with their contemporaries rather than the reverse Gifts of administration, incisive speech, sound judgment, intellectual grasp of difficult subjects, constructive statesmanship, accurate diagnosis of character, great powers of work, untiring energy, quickness of decision, and a masterful spirit may, and often do, compel admiration, but they are apt to excite alarm rather than love. Ernest Hart was a man of undoubted ability and spirit. He possessed the faculty of writing tersely and well; he had a marvellous power of accurately gauging public opinion; was possessed of the true journalistic spirit and instinct, and throughout the twenty-five years of our acquaintance I never knew him at a loss, however sudden the emergency, no matter what difficulties had to be overcome. One strong factor in ministering to the success and influence which he secured was the devoted affection and undeviating loyalty and patience of his sister Charlotte, who possessed much of his ability and many of his gifts. In the old days, when the editor of the *British Medical Journal* had a bandbox of an office over a printer's shop in Great Queen Street, W.C., Miss Hart was ever constant in her attendance, and always in touch with every matter, document, and proof which related in any way to the work of the journal. I believe Miss Hart was the first lady journalist, and she possessed the rare merit of thoroughly understanding her sub-editorial duties, and was always to be found at her post during office hours. Few if any of the present staff of the *British Medical Journal,* who are now splendidly housed in the Strand, can have an idea of the difficulties attending the production of the journal in the days to which we refer. When Mr. Hart became editor the journal was a relatively insignificant publication, with very few readers and on the verge of bankruptcy as a commercial undertaking. By his great journalistic ability he speedily raised the paper to a commanding position, and made it a great literary success. As the journal improved its influence increased, its circulation went up, and so the British Medical Association, from being a small provincial organisation, gradually grew and grew until, thanks mainly to Mr. Hart, it numbers at present some 20,000 members; and the journal has a circulation, not of a few hundreds as formerly, but probably the largest circulation of any medical paper published in the world.

No doubt Ernest Hart had serious faults, and made occasionally bad mistakes; but in justice it must be pointed out that we are all liable to error, and that if the lives of his bitterest enemies were to be fully known, they would probably be found to be the last people in a position to cast a stone at our departed friend, or, for that matter, at any other man amongst their contemporaries. It is by no means a thankful task to be editor to a newspaper which is under the control of a committee formed by members of an association numbering many thousands, every one of whom considers it his privilege to be critical and occasionally hostile and unreasonable. The very conditions of such an organisation must add immensely to the difficulties of editorship. To enable a newspaper to attain a great literary success and to maintain command and influence, it is necessary that its editor shall have a free hand, and that if a free hand is not granted him, he shall have a strong enough personality to take it when occasion demands this sacrifice at his hands, and to defend himself successfully against all and sundry. This Ernest Hart was able to do with almost unvarying success. I therefore claim that on the whole there can be no question that his services as editor of the Journal have been of the very highest order, and that had it not been for him the British Medical Association might have continued a relatively unimportant and uninfluential society.

I have said that Ernest Hart possessed the true instincts of a born journalist. He had an intimate knowledge of the individual members of the medical profession throughout the country, and, indeed, throughout the Empire, and in foreign countries too. He was able to attract to himself every man who could write, and to induce many who had never written, but who possessed sound knowledge, to commence to write, and gradually to acquire the habit of writing, to the no small advantage of the journal, and of the medical profession too. By these means he collected around him as editor the largest staff of writers which probably any journal ever had, so that at any time he was in a position to secure the services of the first authority on any question which came up for discussion. Ernest Hart soon saw that so-called critical reviews of books were very often but the expression of personal enmity to individual writers on the part of those who had no great reason to love the authors, and possibly some reason to belittle their books. He therefore insisted that the duty of a reviewer was to write such a notice as would bring out clearly the contents, aim, and drift of the writer, and then, but not till then, to briefly appraise the value or otherwise of the work under review.

In dealing with such a large number of people it is not surprising that many of them should presume upon the fact that they wrote for the Editor to the extent of expecting that he would treat them with special consideration and even friendship. In other words, every editor soon realises that there are a class of men who write, and who are paid for what they write, but yet consider that the editor is under obligation to them for their contributions. They even feel they have established a claim upon him for special favours and advancement, irrespective of circumstances and, sometimes, even of merit. Hart, as a great editor, never tolerated such liberties, for whilst he was ever ready to co-operate with everybody who had the public interest at heart, and the brains to promote rational schemes for the advantage of the profession or the public, his keen acumen enabled him to explode mere bubbles, to the confusion and annoyance of their parents.

Speaking from intimate knowledge and close association, I am able to declare that in a journalistic experience extending over some thirty years, I have never met a man so just, loyal, and generous as Ernest Hart. We had our differences, and we fought them out like men, but however much we might differ, that never in the end affected our relations as journalists and comrades. I shall never fail to look back with satisfaction upon one such episode, for it proved clearly that Ernest Hart was prepared to recognise his own errors and to make allowances for the errors of other people. If he once took up a cause, or identified himself with an individual, neither the cause nor the individual would ever find him wanting in time of peril or crisis. Such characteristics are as rare as they are honourable to those who possess them. They enable the possessor to write of events and individuals with critical incisiveness and telling effect. Much that Ernest Hart wrote was a model of style, and possibly some of the best work that he ever did was done in 1871 for the *Times* newspaper, when he was entrusted with the difficult task of representing the greatest of English journals during the time of the Prince of Wales's illness at Sandringham in 1871. During all that trying period he never misled the British public. His communications to the *Times* were marked by great ability, clear judgment, and accuracy of diagnosis. Perhaps the most striking of all the articles was one which appeared on the Monday in November following the most critical stage of the Prince's attack, when his life was despaired of. It commenced: "The Prince still lives, and we may still, therefore, hope." It is true the hope that was felt was the near kinsman of fear, but one day more had been wrested from the clutch of death, and in uch a struggle every moment was precious. A book might be written on the subject of the life's work of Ernest Hart, commencing, as it did, when he became associated with the late Dr. James Wakley, of *The Lancet*, during which period he took up and successfully carried through the agitation against the maladministration of the sick wards in workhouses, which resulted in the passing of Hardy's Act and the establishment of the great metropolitan poor law infirmaries, than which few, if any, hospitals are more complete or satisfactory. In later years, under considerable difficulties, he successfully organised and built up the Medical Sickness and Annuity Society, which has already proved a safeguard and a comfort to hundreds of medical men, their wives and families. This society, indeed, is calculated to grow and extend until it embraces every intelligent member of the medical profession. As chairman of the Parliamentary Bills Committee of the British Medical Association, Ernest Hart was the mouthpiece of the medical profession for a prolonged period. On the whole, he used the influence resulting from this important position with singular judgment and marked success. As a journalist, as a contemporary, as a colleague, I found Ernest Hart to be a man whom it was a privilege to know, and one who was able at all times to give more than he ever gained from the majority of the people with whom he became associated. *De mortuis nihil nisi bonum.*

It is to be hoped that the members of the British Medical Association, under the guidance of the Council, will combine as one man to establish some suitable and adequate memorial in recognition of the great services which Ernest Hart has rendered to them individually and collectively, and to the whole medical profession throughout the world.

